# Faecal calprotectin and ultrasonography as non-invasive screening tools for detecting colorectal polyps in children with sporadic rectal bleeding: a prospective study

**DOI:** 10.1186/s13052-020-00828-1

**Published:** 2020-05-20

**Authors:** Giovanni Di Nardo, Francesco Esposito, Chiara Ziparo, Caterina Strisciuglio, Francesca Vassallo, Marco Di Serafino, Maria Pia Villa, Pasquale Parisi, Melania Evangelisti, Claudia Pacchiarotti, Vito Domenico Corleto

**Affiliations:** 1 Chair of Pediatrics, NESMOS Department, Sapienza University of Rome, Sant’Andrea University Hospital, Via di Grottarossa 1035-1039, 00189 Rome, Italy; 2 Pediatric Radiology Unit, Santobono-Pausilipon Children Hospital, Naples, Italy; 3 Department of Woman, Child and General and Specialistic Surgery, University of Campania “Luigi Vanvitelli”, Naples, Italy; 4 General and Emergency Radiology Unit, Antonio Cardarelli Hospital, Naples, Italy; 5 Gastroenterology and Gastrointestinal Endoscopy Unit, Sapienza University of Rome, Sant’Andrea University Hospital, Rome, Italy

**Keywords:** Colorectal polyps, Children, Faecal calprotectin, Ultrasonography

## Abstract

**Background:**

Colorectal polyps are reported in 6,1% of paediatric colonoscopies and in 12% of those performed for lower gastrointestinal bleeding. Although colonoscopy is widely used in paediatric patients, it requires bowel preparation and general anaesthesia or deep sedation, and in rare cases, it can cause complications. Non-invasive screening techniques able to predict polyps in children with isolated and sporadic rectal bleeding may play a key role in the selection of patients needing colonoscopy.

**Methods:**

We enrolled all children undergoing colonoscopy for isolated and sporadic rectal bleeding to determine the diagnostic accuracy of faecal calprotectin, ultrasonography (US) and digital rectal examination as diagnostic methods for screening colorectal polyps.

**Results:**

A total of 26 of 59 enrolled patients (44.1%) had colonic polyps, one patient had multiple polyps, and 23% of children had polyps proximal to the splenic flexure. The diagnostic accuracy of faecal calprotectin for detecting colorectal polyps was 96.6%, with a sensitivity of 100%. False-positive faecal calprotectin was shown in 2 patients with non-steroidal anti-inflammatory drug-related lesions. The diagnostic accuracy of ultrasound was 77.9%. Polyps not seen with ultrasound tended to be relatively smaller (1.5 vs 2.3, *p* = 0.001) and located in the rectum. The combined use of FC, US and digital rectal examination obtained a specificity and PPV of 100%.

**Conclusions:**

FC combined with US and digital rectal examination is a good and promising non-invasive screening test for detecting colorectal polyps in children with isolated and sporadic rectal bleeding.

## Background

Colorectal polyps are reported in 6,1% of paediatric colonoscopies and in 12% of those performed for lower gastrointestinal bleeding. However, these lesions occur much more often than is clinically recognized because many of these polyps simply outgrow their blood supply, become ischaemic, and autoamputate with moderate painless haematochezia [1–3].

Juvenile polyps represent 70–80% of paediatric colonic polyps, and 60–70% of them are solitary. The peak age of diagnosis of juvenile polyps is between 2 and 5 years, with male and non-Caucasian race predominance [4–7].

Lower intestinal bleeding is the most common presenting symptom, but polyps in children often also present with abdominal pain. Bleeding is typically intermittent and self-limited. A history of passing blood mixed with tissue in the stool is often described, and anaemia has been described in 25–35% of patients. Large pedunculated polyps may be pushed distally by peristalsis, leading to intussusception or, in cases of low-rectal polyps, anal prolapse [4, 6, 7].

These polyps are hamartomas and, in contrast to those associated with juvenile polyposis syndrome, are usually not associated with malignant transformation, but they require endoscopic removal to prevent possible sequelae, mainly anaemia and intussusception [4, 7]. However, the natural history of juvenile polyps is unknown, and the exact number of polyps that actually increase cancer risk and the predisposing factors to neoplasia are uncertain. Recent data reveal that solitary polyps in children recur in approximately 17% of cases, and neoplasia may occur in 3.9% [5]. Moreover, adenomatous changes in juvenile polyps, although rare, can still occur, and dysplasia may confer an increased risk of carcinoma [8, 9]. Taken together, these data suggest that current clinical practice to minimize sporadic rectal bleeding and to recommend endoscopic follow-up only if symptoms recur after solitary juvenile polyp removal may be inadequate.

Although colonoscopy is widely used in paediatric patients, it requires bowel preparation and general anaesthesia or deep sedation, and in rare cases, it can cause complications [10]. Non-invasive screening techniques able to predict polyps in children with mild and sporadic symptoms may play a key role in selecting patients needing colonoscopy.

Calprotectin is a non-invasive marker of neutrophilic intestinal inflammation and is markedly elevated in infectious and inflammatory conditions, including Inflammatory Bowel Disease (IBD). It correlates well with histological inflammation and successfully predicts clinical relapses in IBD patients. In addition, it reliably discriminates IBD from functional gastrointestinal disorders because of an excellent NPV for IBD in undiagnosed symptomatic patients [11, 12]. Juvenile polyps are composed of inflammatory cells, including neutrophils, and the mucosal surface is often very friable. Degradation and exfoliation of neutrophils into the stool result in increased levels of faecal calprotectin [4, 13, 14]. Recently, colonic juvenile polyps have been associated with increased levels of faecal calprotectin (FC) comparable to those observed in active inflammatory bowel disease (IBD), suggesting its potential role as a non-invasive biomarker able to direct endoscopic evaluation [13, 14].

Ultrasonography (US) has emerged as an accurate non-invasive tool for detecting colorectal polyps with high reported specificity but with low sensitivity [15–21]. The combined use of these two potentially complementary non-invasive tools has never been reported in children with suspected colonic polyps.

The aims of this prospective study are to determine the diagnostic accuracy of faecal calprotectin, abdominal US and digital rectal examination as a primary diagnostic method to select patients needing colonoscopy among children with isolated and sporadic rectal bleeding. Clinical, endoscopic and histological findings of colorectal polyps in children will also be evaluated.

## Methods

We prospectively enrolled all 1–18-year-old patients referred to the Pediatric Gastroenterology Unit of Santobono-Pausilipon Children’s Hospital of Naples from June 2017 to April 2019 who underwent colonoscopy due to sporadic lower gastrointestinal bleeding (defined as no more than 3 episodes of rectal bleeding with normal stools in the last year). Only patients with isolated lower gastrointestinal bleeding were included in our study. Children with characteristics of inflammatory bowel disease such as diarrhoea, arthritis, perianal disease, weight loss, or increased serum inflammatory markers were excluded. In addition, patients with known polyposis syndrome or other underlying diseases that may affect the bowel, such as graft versus host disease, were also excluded. Abdominal pain and constipation (without an apparent anal fissure) were considered nonspecific symptoms and were not excluded.

For each enrolled patient, we collected demographic data, clinical presentations, symptom durations, laboratory test results, faecal calprotectin levels, US findings, colonoscopy findings and histology results.

Ileocolonoscopy was performed with a paediatric colonoscope (Olympus PCF-H190/I, Hamburg, Germany) under general anaesthesia or deep sedation with propofol after adequate bowel cleansing [22]. Colonoscopy findings including polyp location, number, size, morphology and removal technique were registered. Polypectomy was performed with a standard polypectomy snare using different prophylactic techniques, such as injection of an epinephrine saline solution in the stalk or a detachable loop or clip positioned over the stalk according to the polyp size and location. When technically feasible, polyps were retrieved for histological evaluation.

A calprotectin assay was performed within a month before the colonoscopy in all enrolled patients using a commercially available enzyme-linked immunosorbent assay test (Calprest Eurospital, Trieste, Italy) and was repeated after 1 month in patients who underwent polypectomy and in patients with positive FC at baseline. According to the manufacturer, calprotectin levels exceeding 100 mg/kg were considered positive.

An abdominal US scan, without any colon preparation or sedation, was performed in all enrolled patients within a week before the colonoscopy. To reduce the amount of food and air in the small bowel, a fasting period of at least 4 h was recommended. US was performed by two experienced radiologists (FE and MDS) using both a low-frequency convex transducer and a high-frequency linear transducer (range of frequency 9–15 MHz, MyLabe Twice, Esaote, Genova - Italy) adopting a standardized colonic ultrasound investigation. The latter consisted of transverse and longitudinal sections for each segment according to European Federation of Societies for Ultrasound in Medicine and Biology (EFSUMB) advice [15]. First, the caecum and the ascending colon were identified in the right quadrant of the abdomen. Then, the colon was studied from the right colonic flexure along the transverse colon to the splenic flexure. The descending colon was finally identified by its latero-dorsal position and scanned caudally to the sigmoid colon, which takes a variable course over the left iliac vessels to the small pelvis. The rectum was visualized through the filled bladder. Two images (longitudinal and axial) were usually acquired for each part of the rectum, sigmoid colon, descending colon, transverse colon and ascending colon using a linear transducer. Colour Doppler ultrasound was used as needed. Colorectal polyps were diagnosed based on criteria established by previous reports, such as the presence of a rounded hypoechoic nodule located within the colonic lumen and with a peripheral hyperechoic layer, sometimes containing small cysts, attached to the intestinal wall through a peduncle with arterial and venous blood flow in colour Doppler mode [15–21]. US findings including polyp location, number, size and morphology were collected.

This study was approved by the Ethical Committee of Santobono-Cardarelli Hospitals and was performed according to the principles of the Declaration of Helsinki, and written informed consent was obtained for all study participants from a parent or legal guardian.

### Statistical analysis

**Fig. 1:**
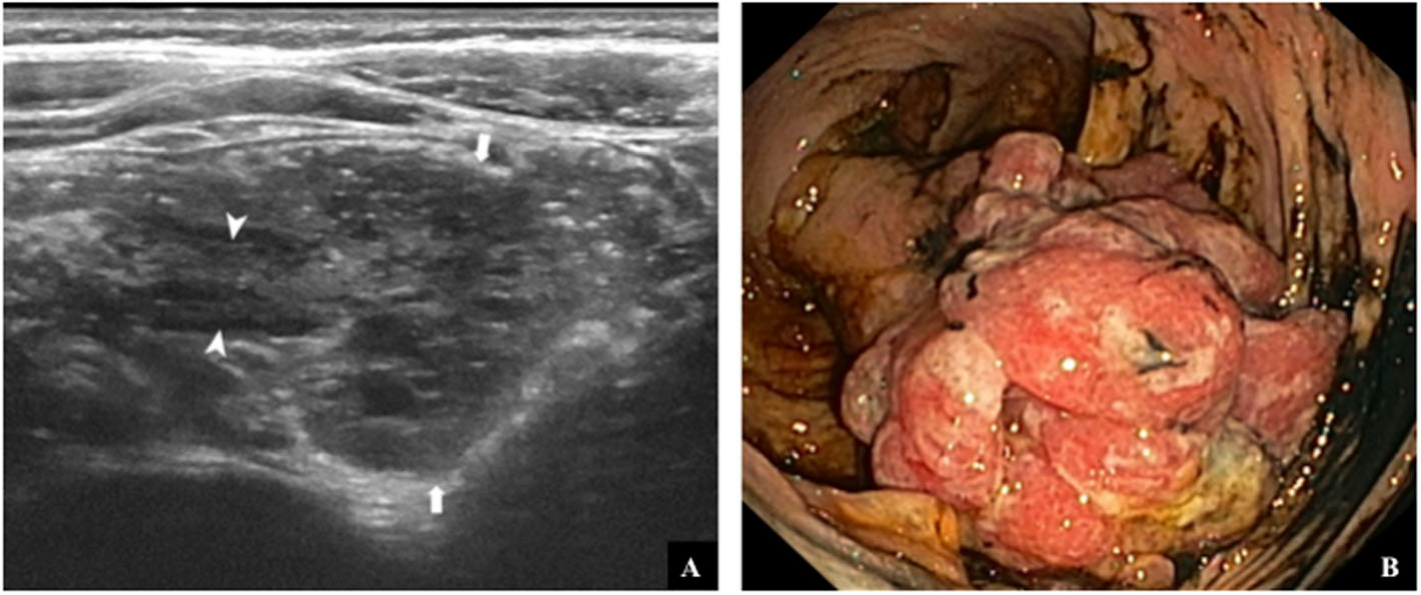
US (**a**) and endoscopic (**b**) appearance of right colonic polyp. US shows a rounded mass containing several tiny cyst (arrows). Graded compression demonstrates a pedicle (arrowheads) that ties the polyp to the right colon wall

The normal distribution of data was assessed by means of the Kolmogorov-Smirnov test. Continuous variables were expressed as arithmetic means ± SDs or medians (IQRs) depending on their distribution. Pearson’s or Spearman’s correlations were used to test the relationship between continuous variables, as required. Contingency tables (Chi-square test with Fisher’s correction) were used to compare categorical variables, and independent t-tests or Mann-Whitney tests were used for continuous variables according to the normal distribution of the data. We calculated the sensitivity, specificity, positive predictive value, negative predictive value and accuracy of digital rectal examination, faecal calprotectin and abdominal ultrasound, alone and combined, in the diagnosis of colorectal polyps. SPSS software (version 19, SPSS Inc., Chicago Il, USA) was used for the analyses. “P” values of < 0.05 were considered to be statistically significant (Fig. 1).

## Results

A total of 174 patients referred for colonoscopy due to lower gastrointestinal bleeding were evaluated. Of them, 115 patients were excluded due to other complaints in addition to lower gastrointestinal bleeding and for the following reasons: suspected IBD (31), clinical relapse [19] or follow-up with known IBD [23], polyposis syndrome [23], allergic colitis [8], and Meckel’s diverticulum [7].

**Fig. 2:**
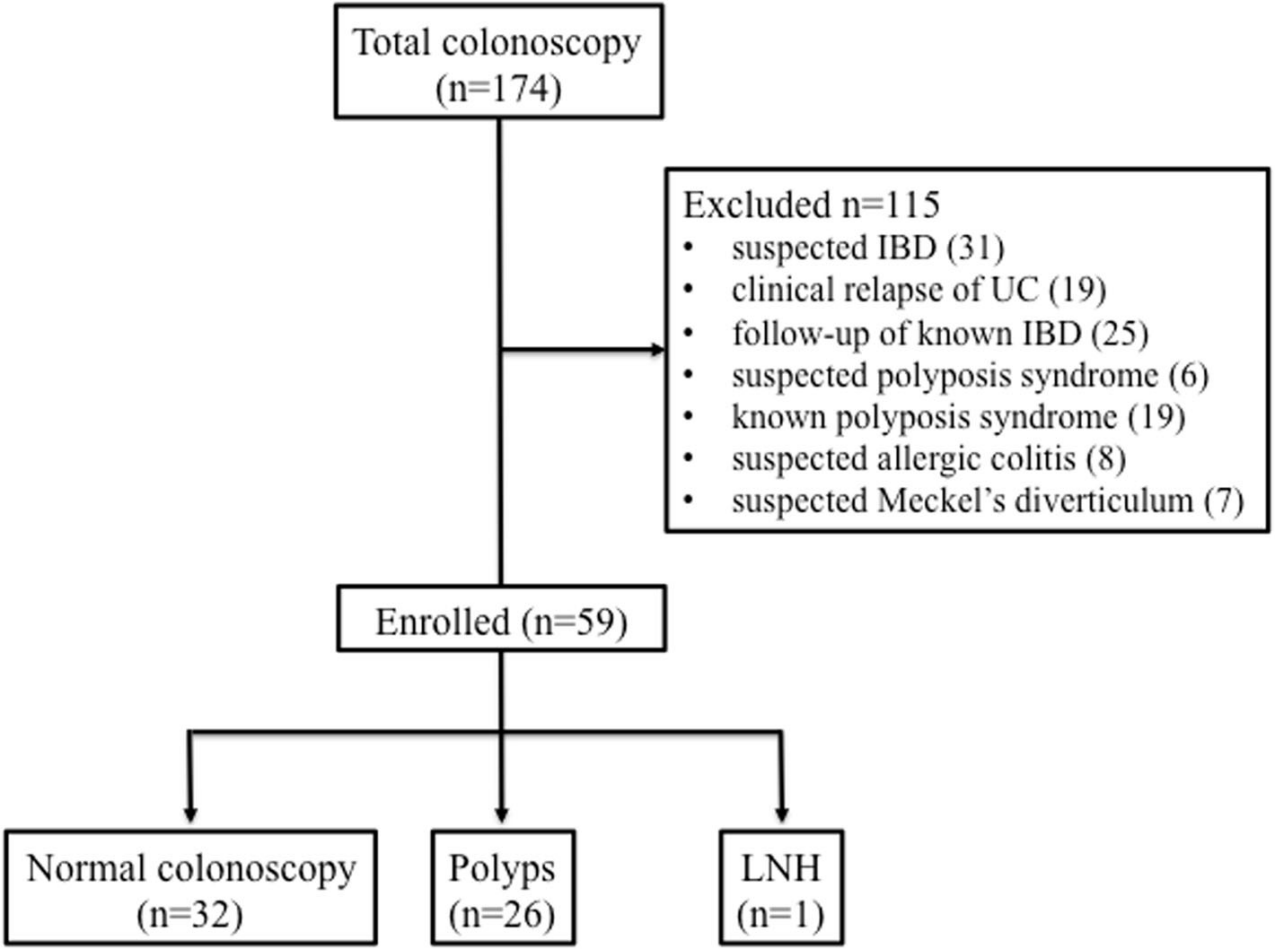
Study flow

A total of 59 patients met our inclusion criteria. The median age at presentation was 7.0 years (age range 2–15 years), and 31 patients (52.5%) were male. The study flow is summarized in Fig. 2.

The main demographic and clinical features of the enrolled patients according to the presence or absence of polyps are summarized in Table 1. There was a male predominance and a prevalence of anaemia and tenesmus in patients with polyps, without reaching significant differences when compared with children without polyps. Normal colonoscopy findings and ileocolonic lymphoid nodular hyperplasia were observed in 32 (54%) and 1 (1.6%) patient, respectively.

**Table 1:** Demographics and clinical characteristics of the enrolled patients according to the presence or absence of polyps

	**Patient with polyps (*n*** **= 26)**	**Patient without polyps (*n*** **= 33)**	***p*-value**
Age; median (range)	5.0 yrs. (2–15)	7.0 yrs. (4–15)	NS
Male; n (%)	15 (57.7%)	16 (48.5%)	NS
Anemia; n (%)	1 (3.8%)	0	NS
Symptoms duration; median (range)	7.8 mo (2–30)	6.0 mo (3–18)	NS
Stool pattern; n (%)			
• Normal	23 (88.5%)	27 (81.8%)	NS
• Constipation	2 (7.7%)	6 (18.2%)	NS
• Diarrhoea	1 (3.8%)	0	NS
Abdominal pain; n (%)	3 (11.5%)	3 (9.1%)	NS
Tenesmus; n (%)	4 (15.4%)	0	NS

Continuous variables are expressed as arithmetic means ± SD or median (interquartile range)

Mann–Whitney U test, independent T test or Chi square test were used when appropriate

*NS* Not significant

Twenty-six out of 59 enrolled patients (44.1%) had colonic polyps. The main endoscopic, histological and detailed endoscopic removal techniques are summarized in Table 2.

**Table 2:** Endoscopic and histological characteristics of detected polyps

**Variables**	
Patients with polyps; n (%)	26 (44.1%)
Patients with single polyps; n (%)	25 (96.2%)
Patients with multiple polyps; n (%)	1 (3.8%)
Polyps distribution and frequency; n (%)	
• Total number of polyps	27
• Right colon	0
• Transverse colon	6 (22.2%)
• Left colon	12 (44.4%)
• Rectum	9 (33.3%)
Polyp size; median (range)	1.7 cm (range 1–4.3 cm)
Morphology; n (%)	
• Pedunculated	27 (100%)
• Sessile	0
Endoscopic removal technique; n (%)	
• Epinephrine saline solution	20 (74.1%)
• clip	6 (22.2%)
• detachable loop	1 (3.7%)
Complications; n (%)	0

A total of 27 polyps were detected at colonoscopy in 26 children: 6 (22.2%) in the transverse colon, 12 (44.4%) in the left colon and 9 (33.3%) in the rectum. A total of 23% of children had polyps proximal to the splenic flexure. Single polyps were found in 25 (96.1%) patients. The median polyp size was 1.7 cm (range 1–4.3 cm).

**Fig. 3:**
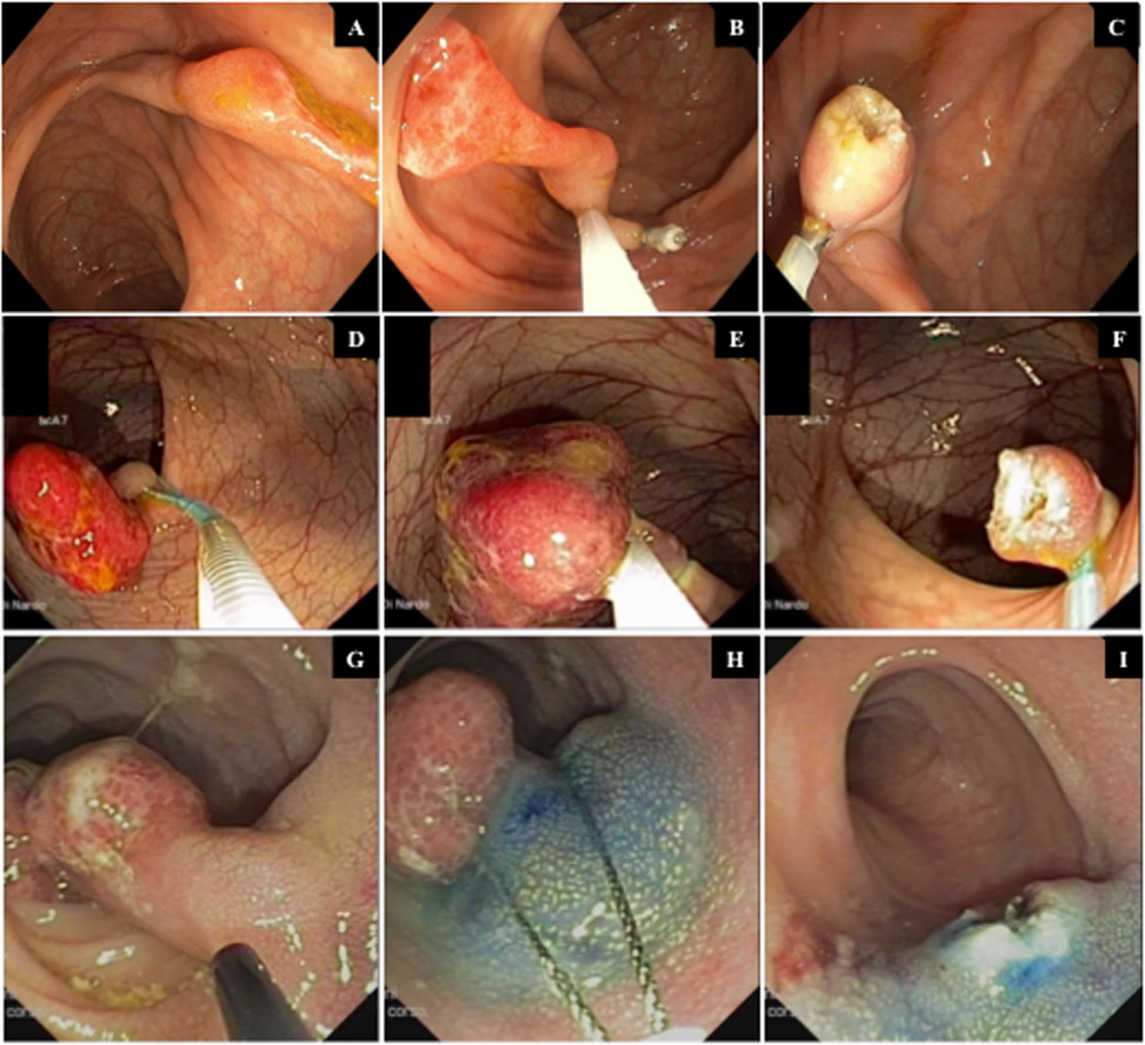
Endoscopic findings of three polyps removed with standard polypectomy snare using different types of prophylactic methods to prevent post-polypectomy complications: clip placement (**a**,**b**,**c**) and detachable loop (**d**,**e**,**f**) positioned over the stalk or injection of an epinephrine saline solution in the stalk (**g**,**h**,**i**)

All polyps were successfully removed with a polypectomy snare after prophylactic injection of an epinephrine saline solution (1:20000) in the stalk, with a clip or with detachable loop placement over the stalk in 20 (74.4%) cases, 6 (22.2%) cases and 1 (3.7%) case, respectively (Fig. 3). No post-polypectomy complications occurred, and all polyps were retrieved for histological analysis and resulted in juvenile polyps according to histology.

Polyps were palpable during digital rectal examination in 77.8% of rectal polyps, accounting for 25.9% of all diagnosed polyps; in one case, rectal polyps prolapsed from the anus.

Of the 26 children with colorectal polyps, ultrasound detected polyps in 13 (50%) children. US detected polyps in 4 out of the 6 patients (66.6%) with transverse colon polyps, 9 out of the 11 patients (83.3%) with left colon polyps and 0 out of 9 patients (0%) with rectal polyps.

The differences between polyps detected and undetected by US are reported in Table 3. When ultrasound showed false-negative results, polyps tended to be smaller (1.5 vs 2.3, *p* = 0.001) and located in the rectum; in contrast, the US detection rate was significantly higher in the left colon.

**Table 3:** Differences between polyps detected or not detected with US

	**Polyps detected by US (*n*** **= 14)**	**Polyps not detected by US (*n*** **= 13)**	***p*-value**
Age; median (range)	4.6 (2–15)	6 (2–12)	NS
Male; n (%)	6 (42.8%)	9 (64.3%)	NS
Anemia; n (%)	1 (7.1%)	0	NS
Calprotectin levels; median (range)	688 (264–2736)	550 (220–1400)	NS
Polyp maximum diameter; median (range)	2.3 (1.5–4.3) cm	1.5 (1–1.8) cm	0.001
Polyps location			
• Right colon	0	0	NS
• Trasverse colon	4	2	NS
• Left colon	10	2	0.001
• `Rectum	0	9	0.001

Mann–Whitney U test or Chi square test were used when appropriate

*NS* Not significant

Faecal calprotectin levels were positive (median 685 mg/kg; range 220–2736) in all patients (100%) with polyps and in 2 out of 33 patients (6%) without polyps. The two patients without polyps and with positive faecal calprotectin levels had non-steroidal anti-inflammatory drug (NSAID)-related false-positive calprotectin. One month after polypectomy and NSAID interruption, the faecal calprotectin levels returned to the normal range.

The sensitivity, specificity, positive predictive value (PPV), negative predictive value (NPV) and accuracy of digital rectal examination (DRE), faecal calprotectin and abdominal ultrasound for the diagnosis of colorectal polyps, alone and combined, are summarized in Table 4.

**Table 4:** Performance of digital rectal examination, fecal calprotectin and abdominal ultrasound for detecting colorectal polyps

**Technique**	**SE, % (95% CI)**	**SP, % (95% CI)**	**PPV, % (95% CI)**	**NPV, % (95% CI)**	**ACC, %**
**Digital rectal examination (DRE)**					
• **Overall**	26.9% (9–44)	100%	100%	63.5% (59–67)	67.8%
• **Rectum**	77.8% (51–100)	100%	100%	94.2% (86–100)	95.2%
**Fecal calprotectin (FC)**	100%	93.9% (72–100)	92.8% (84–100)	100%	96.6%
**Abdominal ultrasound (AUS)**	50% (31–69)	100%	100%	71.7% (59–84)	77.9%
**Combination of FC and AUS**	86.6% (75–98)	100%	100%	67.4% (54–80)	74.6%
**Combination of FC and DRE**	33% (16–50)	100%	100%	59.6% (46–73)	64.4%
**Combination of AUS and DRE**	53.8% (27–81)	100%	100%	88.4% (71–100)	89.8%

*Abbreviations: SE* Sensitivity, *SP* Specificity, *PPV* Positive predictive value, *NPV* Negative predictive value, *ACC* Accuracy, *CI* Confidence interval

## Discussion

The present study showed a 14.9% prevalence of colorectal polyps in children undergoing colonoscopy for lower gastrointestinal bleeding. Associated symptoms included abdominal pain (11.5%) and tenesmus (15.4%), and in one case, polyps prolapsed from the anus. However, these data are similar to those previously reported [1].

Although polyps were usually solitary and located in the left colon or rectum in our series, 23% of children had polyps proximal to the splenic flexure. These data, in agreement with a previously published study [23–27], support the need for total colonoscopy in all children with suspected colonic polyps and recurrent painless rectal bleeding.

In contrast with previous reported series [21], we were able to perform polypectomy in all patients without complications, and this result can probably be explained by the routine use of prophylactic methods before the standard polypectomy technique.

In our study, polyps were detected with digital examination in 25.9% of all children with colorectal polyps and in 77.8% of those with rectal polyps. According to previous reports [21, 23, 26], we confirm the significance of digital examination to detect rectal polyps and to exclude other rectal causes of bleeding (i.e., anal fissure, intussusception and rectal prolapse).

Many children with colorectal polyps have subtle symptoms with moderate isolated and sporadic rectal bleeding and are thus expected to remain undiagnosed because of insufficient investigations. Affordable strategies for detecting colorectal polyps are therefore still needed, especially in children in whom the symptoms and clinical signs can be difficult to distinguish from more common functional or self-limiting gastrointestinal conditions, wherein invasive methods such as colonoscopy should be avoided.

Recent studies, due to the safety, acceptance and accuracy, suggest US as a primary diagnostic method to screen children for colorectal polyps before colonoscopy [16–21]. In this study, we describe the diagnostic performance of US, without colon preparation, for detecting colorectal polyps. The sensitivity, specificity, PPV, NPV and accuracy of the procedure were 50, 100, 100, 71.7 and 77.9%, respectively. In the case of false-negative results on US, the polyps tended to be relatively smaller and located in the rectum. These data are in accordance with a previously published study and are clinically relevant in managing paediatric patients with suspected colorectal polyps because the rectum and sigmoid colon are the most common sites for colorectal polyps [1, 4, 13, 14]. Many factors can affect the US detection of colorectal polyps: operator skills, polyps located deep inside the pelvis, and the amount of faeces in the rectum [4, 16]. Indeed, a limitation of our study is that US was performed without colon preparation. Qu et al. reported that colorectal polyps located in the sigmoid colon or rectum were detected by US only in 65% of cases without colon preparation, whereas after glycerine enema detection, the rate increased to 97%. Therefore, when colorectal polyps are suspected, colon cleansing before US should be recommended [17].

We showed that all children with juvenile polyps had elevated FC levels, which always normalized after polypectomy. These results, in accordance with the study from Olafsdottir et al. [14], suggest that calprotectin can also be a useful tool for the follow-up of children with a previous history of colorectal polyps; however, we also acknowledge that specific studies are needed to address this issue. In our study, the sensitivity, specificity, PPV, NPV and accuracy of FC for detecting colorectal polyps were 100, 93.9, 92.8, 100 and 96.6%, respectively. In the case of false-positive FC levels, 2 patients had NSAID-related lesions. A previously published study reported similar results [13, 14]. However, the combined use of FC, US and DRE obtained a specificity and PPV of 100%.

## Conclusions

Although these data need to be confirmed in larger multicentric studies, our study shows that FC combined with US and DRE has a very high PPV and specificity. Therefore, these approaches can be considered good non-invasive screening tests for detecting colorectal polyps in children with isolated and sporadic rectal bleeding.

## Data Availability

Complete data are available at Santobono-Pausilipon Children’s Hospital.

## Abbreviations

IBD: Inflammatory Bowel Disease
FC: Fecal Calprotectin
NPV: Negative Predictive Value
NSAIDs: Non-Steroidal Anti-Inflammatory Drugs
US: Ultrasonography
PPV: Positive Predictive Value
